# Service Area Delimitation of Fire Stations with Fire Risk Analysis: Implementation and Case Study

**DOI:** 10.3390/ijerph17062030

**Published:** 2020-03-19

**Authors:** Wenhao Yu, Yujie Chen, Zhanlong Chen, Zelong Xia, Qi Zhou

**Affiliations:** 1 School of Geography and Information Engineering, China University of Geosciences, Wuhan 430074, China; chenzl@cug.edu.cn (Z.C.); sevenlaand@hotmail.com (Q.Z.); 2 State Key Laboratory of Resources and Environmental Information System, Institute of Geographical Sciences and Natural Resources Research, Chinese Academy of Sciences, Beijing 100101, China; 3 Key Laboratory of Geological Survey and Evaluation of Ministry of Education, China University of Geosciences, Wuhan 430074, China; 4 School of Earth Sciences and Engineering, Hohai University, Nanjing 210098, China; 160211020003@hhu.edu.cn

**Keywords:** fire stations, fire incident, service area, emergency facilities

## Abstract

Under the rapid development of urbanization, fire service becomes one of the biggest contributive factors to personal health and property safety. A reasonable plan of fire services should first address the issue of service area delimitation for fire emergency facilities. Specifically, there are two key factors for fire services including rescue efficiency and load balancing, which are usually handled by the space partitioning methods (e.g., Voronoi diagram). The traditional methods tend to model the space in a homogeneous plane with Euclidean distance, while in reality, the movement of rescuing is constrained by the street network. In addition, the built environment is complex by its variation of fire risk across places. Therefore, we propose a novel constrained Voronoi diagram for fire service area delimitation by adding the datasets of street network and historical fire incidents. Considering the prior knowledge that a fire engine is expected to reach the location of incident within five minutes, which is also called Golden 5 min, we propose a network partitioning algorithm which is able to increase the five-minute coverage of fire stations. Through a case study in Nanjing, China, we demonstrate the practicability of the proposed method in delimitating service areas of fire stations across time.

## 1. Introduction

The demarcation of service area is one of the most important issues in spatial partitioning, as a reasonable service area delimitation always increases the efficiency of facilities service. It has been widely studied in various fields, for example, trade zones planning [1,2,3,4,5], medical services area planning [6,7,8], and police patrol areas planning [9,10,11,12]. Although many methods have been developed to deal with spatial partitioning and service area delimitation problems, it remains a challenging task when establishing a spatial proximity model independent of the coordinate geometry.

Voronoi diagrams are widely used to deal with spatial optimization and spatial partitioning problems, especially in delimiting the service area of facilities [13,14,15,16]. Recently, Qiao et al. [17] used the Voronoi diagram to create service areas for the charging stations in Shanghai and then identified the one with the highest time cost to locate the new facility. Despite the planning of urban services being extensively studied in the past, the balancing between service demand and supply remains a challenging task in the new complex environment. In the rapid development of urbanization, the unbalanced built environment leads to the spatial heterogeneity of service demand. At the same time, the intricate roads have greatly affected the ability of urban services. Therefore, the traditional Voronoi diagram that models the urban space as a homogeneous environment is no longer suitable for delimiting the service areas of current urban facilities.

In the literature, many extensions of the Voronoi diagram have been developed to adapt to the complexity of real-world geographic scenes. For example, Okabe et al. [18] formulate six Voronoi diagrams defined on the network, termed generalized network Voronoi diagrams, to precisely represent service areas in urban areas. The extension of the Voronoi diagram to a network space is more in line with the real characteristics of a street network service transmission and more suitable for urban activities, e.g., retail services. With considering the geographic distribution of demands and the socioeconomic context, Wang et al. [19] apply the hexagon-based adaptive crystal growth Voronoi diagrams to delimit middle school service areas. The results indicate that, compared with the raster-based method, the hexagon grid method is more accurate in measuring continuous weighted planes. Furthermore, the weighted Voronoi diagram allows for a more detailed modeling of services by introducing the varied capabilities of a facility. It is widely used in urban planning applications including trade zone design [20], transportation and logistics problems [21,22,23], political districting [24], substation planning [25,26], and school districting [19,27].

However, there are few studies on the service division applied to urban emergency facilities, especially fire stations. As a critical emergency facility in cities, a fire station aims at providing timely services to cover nearby fire incidents and associated events. However, the current emergency system in China delimits the service areas of fire stations based mainly on a 2D polygon, such as block units, which is hard to take into account the variation of fire risk across regions. In addition, the existing service areas of fire stations ignore the actual route of rescue vehicles, which may consume a large amount of rescue time. Therefore, a reasonable division of fire station service area should consider three aspects including rescue accessibility, balanced workload, and related legal requirements.

Under the context of fire rescue, fire engines reach the locations of incidents through the route of a street network, and in practical applications, the main attention of planners is also paid to the division of network segments for understanding the accessibility of fire emergency service. Hence, it may be more appropriate to define the service areas of fire stations using the constraint of the street network. Besides rescue accessibility, the constraint of balanced workload should also be considered in the region partitioning method. According to the studies in resource allocation [28], the unbalanced workload could lead to some areas becoming overstaffed while others become understaffed. It is believed that the unbalanced workload can even lead to morale problems and span-of-control issues. In this paper, we quantify the workload by the density of events. Kernel density estimation (KDE) is a popular method in analyzing the first-order properties of point event distributions [29,30,31]. KDE generates smooth density surfaces of point events in space by calculating the intensity of events as density estimation. With much attention paid to the network analysis, network kernel density estimation was also used in analyzing urban point events, e.g., traffic accidents [32].

Furthermore, according to the regulations of the Ministry of Public Security, an important principle of fire station planning is that fire engines can reach the locations of fire events in 5 min [33]. Such Golden 5 min constraint can ensure that the fire damage is efficiently controlled by the fire fighters before residents suffer a great loss in the fire. However, the constraint of workload requires assigning a place to a fire station according to fire risk, and there is a need to improve the Voronoi model by increasing the cumulative coverage area of 5 min of all the Voronoi generators.

With the abovementioned three issues considered, we propose a constrained Voronoi diagram (C-VD) to detect the service area for emergency facilities (e.g., fire station). In general, our method is more effective than the traditional method by introducing three constraints including street network constraint, fire risk constraint, and Golden 5 min fire rescuing constraint. More specifically, considering that the interaction between demand and supply of fire service is along the street network, we adopt the network shortest-path distance in proximity analysis and obtain the service area of fire stations as the network section consisting of segments and nodes. In addition, since the fire risk varies across locations of urban space, the regions with the same area may have different workloads in the emergency service system, and thus, we propose to weigh the network distance by quantifying the workload of locations on the street network. This process is realized by adding the data of historical fire incidents. Furthermore, in order to increase the cumulative 5-min coverage area of fire stations, we propose a new algorithm for generating a C-VD. Specifically, all the generators of the fire station expand along the street network simultaneously, and when an operator reaches the boundaries of the 5-min neighborhood, it stops until all the other operators meet the time threshold of 5 min. Our algorithm introduces the fire risk into distance calculating, and at the same time, it takes into account the specific requirement of fire rescuing, i.e., increasing the cumulative coverage area of 5 min of fire stations. In this way, the space tessellation of a C-VD can be very different from the result of the general Voronoi diagram.

The paper is organized as follows: Section 2 details the methodology of C-VD. Section 3 describes the study area and data. Section 4 analyzes and discusses the experimental results. Section 5 concludes the research and provides future directions.

## 2. Methodology

Although our proposed C-VD is still based on the principle of assigning a place to the nearest generator, it introduces three additional constraints (i.e., street network constraint, fire risk constraint, and Golden 5 min constraint) to obtain a more practical result of the service area division for fire emergency facilities. To incorporate all the constraints, we first project fire stations to the nearest locations on the street network and define their service area as a set of network segments and nodes (Section 2.1). In this way, the fire station provides emergency service along the network distance rather than Euclidean distance. Second, the fire risk on network segments is quantified by using the kernel density analysis of historical fire incidents, and then, the network distance is weighted by such a variable to calculate the workload on each network segment (Section 2.2). Finally, an extended network expanding algorithm is used to help increase the 5-min coverage areas of C-VD of the fire stations (Section 2.3).

### 2.1. Constraint of Street Network

In order to model the constraint of network distance, we use a graph structure to represent the street network. Specifically, a street network is formalized as G=(V,E) where V is the set of nodes and E is the set of edges in the street network. For simplicity of concept demonstration, we assume the graph G is planar and non-directed.

**Figure 1:**
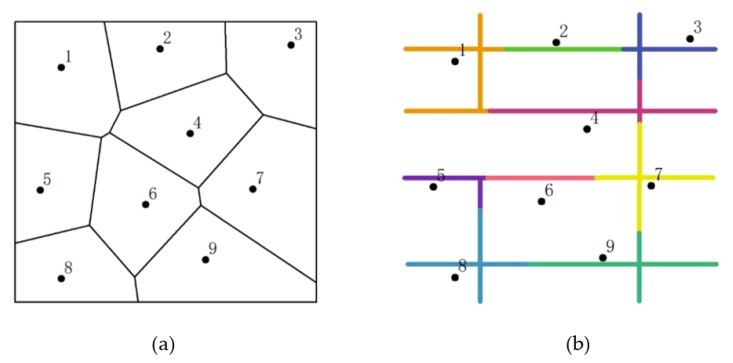
The comparison between two partitioning methods: (**a**) the traditional Voronoi diagram based on planar partitioning and (**b**) the street-based partitioning model.

In addition, the expanding operation of generator points in the C-VD is carried out along the street network, and thus, the next step is to project the generator points $\;S=\left\{S_{1},S_{2}\ldots S_{n}\;\right\}$ onto their corresponding nearest locations on the street network. This process can be realized by calculating the Euclidean distance between generator points (i.e., locations of fire stations) and network segments. At the end of this stage, the projected points on the network are detected as the proxy of fire stations for further analysis. Figure 1 presents a comparison between the traditional Voronoi diagram and our division method with adding street network restriction.

### 2.2. Constraint of Fire Risk

In addition to the geometric distance, fire risk is another important factor that constrains the service area of a fire station by representing the demand in urban space. In general, the more likely the fire events could occur, the larger workload the neighboring fire stations have. Hence, it is critical to quantify the distribution of fire risk across the space. Actually, there are many factors contributing to fire risk, e.g., population age, building age, building density, land use, etc. The result of fire risk variation can be directly reflected by the density patterns of historical fire event points. The more fire incidents a local region has, the higher fire risk it has and, then, the larger the workload covered for it should be. In addition, fire risk should be distributed around the location of the fire incident. More specifically, the actual location where a fire incident occurs has the largest risk, and with the increase of distance from the incident location, the neighboring locations have a decreased risk. This conforms to the requirement of a “distance decay effect”, which is a specific form of the First Law of Geography in spatial analysis research. Taking into account these factors, we adopt the kernel density method to calculate the fire risk in space.

**Figure 2:**
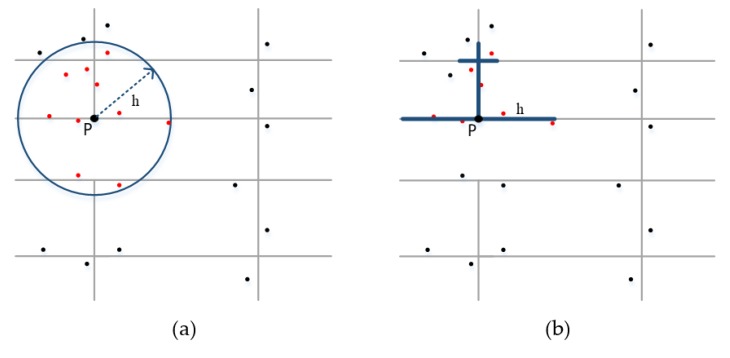
The comparison between planar kernel density method (**a**) and network kernel density method (**b**): The point P represents a sample point on the street network; h is the bandwidth; and dot points represent events, among which red dots represent events that fall within the catchment of P.

The kernel density estimation (KDE) method uses a kernel function to model the “distance decay effect”, in which the point density value decreases gradually with increasing distance from the focus. Many studies have proved that the network kernel density estimation (N-KDE) is more suitable for analyzing hot spots occurred inside the network space than traditional planar KDE [32]. They suggest that the planar KDE (Figure 2a) could possibly over-detect clustering patterns [32].

Therefore, we propose to use the N-KDE to calculate the spatial density values of fire incidents on the street network (Figure 2b). In order to obtain the kernel density distribution of the entire road network, we need to split all the links in the network by a predefined segment size. The link segmentation has been a common practice for network-based analyses that need to capture the distribution of events across street network [30,31,32,34]. Therefore, we firstly divide each street segment into basic linear units of a predefined network length, and then, for a point $p_{j}$ on the network, its kernel density value $f\left(p_{j}\right)$ is given by the following:

(1) $$f\left(p_{j}\right)=\overset{n}{\underset{i=1}{\sum }}\frac{1}{h^{2}}K\left(\frac{p_{j}-e_{i}}{h}\right)$$

where n is the total number of event points within the range of network distance threshold h, h is the search bandwidth, $e_{i}\;$ is the projection point of event located in the catchment of $p_{j}$, $p_{j}-e_{i}$ is the network distance between point $p_{j}$ and point $e_{i}$, and K is the kernel function that models the “distance decay effect” of the density. In the literature, there are many forms of the kernel function such as the Gaussian, Quartic, and Conic. Although these functions weigh the density values in different forms, they are all based on the same process that the weight value decays from the center to the surrounding locations according to distance. In addition, it has been widely accepted that the choice of function is less important than the choice of an appropriate search bandwidth (i.e., catchment size) [31,32]. Therefore, this paper chooses a quadratic function (Equation (2)), which is one of the most commonly used functions in spatial analysis.

(2) $$K\left(\frac{p_{j}-e_{i}}{h}\right)=\frac{3}{4}\left(1-\frac{{\left(p_{j}-e_{i}\right)}^{2}}{h^{2}}\right)$$

It should be noted that there is no standard method to determine the bandwidth and the threshold should be adjusted according to the application context [31]. In general, a large bandwidth emphasizes the global pattern of point distribution while a small bandwidth can help detect the detailed variations of point pattern. Our research determines the bandwidth according to the specific context of fire emergency service (please see Section 4.1).

**Figure 3:**
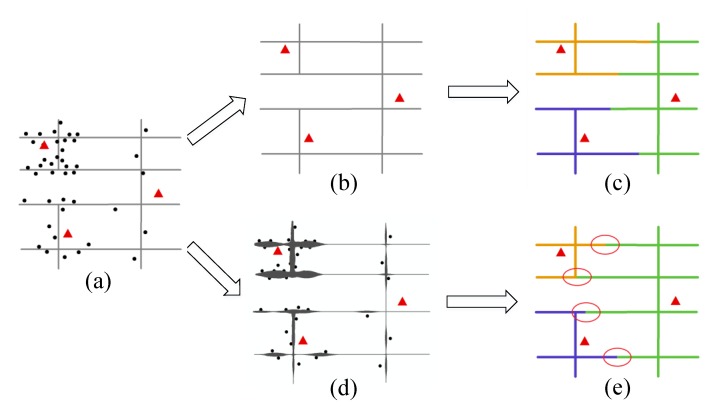
The generating process of the Voronoi diagram with (or without) considering fire risk variation across space: (**a**) the original data of fire stations (red symbols), fire accidents (black symbols), and street network (black lines); (**b**,**c**) the Voronoi diagram constructed by considering only the geometric length of street segment; (**d**) the fire risk calculated by network kernel density estimation of fire accidents; and (**e**) the constrained Voronoi diagram constructed by considering both the geometric length and fire risk.

In order to integrate the two indicators (i.e., geometric distance and fire risk) into the cost ($W_{i}$) of a street segment, we use the weighting method as follows.

(3) $$W_{i}=a\cdot \frac{L_{i}}{\sum^{\text{​}}L}+\left(1-a\right)\cdot \frac{R_{i}}{\sum^{\text{​}}R}$$

where $L=\left\{L_{1},L_{2}\ldots L_{n}\;\right\}$ and $R=\left\{R_{1},R_{2\ldots }R_{n}\;\right\}$ respectively represent the geometric length and the fire risk of each segment in the street network and where  a is a factor that determines the weight of the geometric length in integration. In the practical applications, the parameter a represents the degree to which users attach importance to the constraint of geometric distance. Some applications emphasize the influence of distance on workload, while others may turn to the fire risk. In general, the contribution of the indicators should be determined under the specific application context, and in our research, we would evaluate the behavior of a C-VD with the change of the weight *a* (please see Section 4.1). An example is presented in Figure 3, where the VDs with considering fire risk and without considering fire risk are compared. It can be found that, after adding the fire risk constraint, the fire stations having frequent fire accidents in their neighborhoods have a reduced service area, and that means the workloads are balanced via reducing the geometric coverage in our model (Figure 3).

### 2.3. Implementation

The procedure of generating a network-constrained Voronoi diagram is based on the network expansion operation, which starts from the locations of the generator points and expands outwards along the network gradually and synchronously. During this process, the node that has the minimum cost in the current iteration will be assigned to the service area of the corresponding generator. It should be noted that the general operation of network expansion does not take into account the specific requirement of fire rescuing (i.e., Golden 5 min constraint): that is, the fire engine should reach the location of the fire incident within 5 minutes. Hence, we propose a new algorithm to increase the 5-min coverage area of the general Voronoi diagram. According to the official report by the Fire Department of Ministry of Public Security, the average speed of urban fire truck is about 30–35 km/h. In addition, we analyzed the GPS track data of fire trucks in Nanjing and found that the average speed of fire trucks was 32 km/h. Therefore, the time threshold of 5 min in our method can be converted into the geometric distance of about 2667 m.

**Figure 4:**
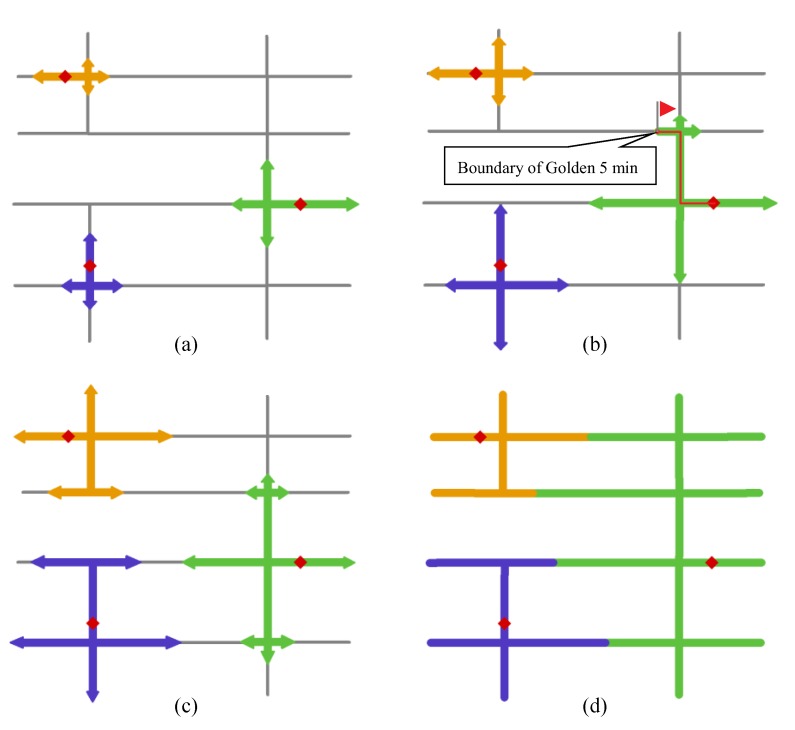
The implementing of the constrained Voronoi diagram: (**a**) expanding along the network simultaneously from the locations of fire stations, (**b**) stopping the operator that reaches the boundaries of Golden 5 min coverage and continuously expanding the others, (**c**) re-executing the operators synchronously when all of them reach the boundaries of Golden 5 min coverage, and (**d**) obtaining the final result.

Based on the context mentioned above, the following steps are implemented to generate the proposed C-VD with the Golden 5 min constraint (Figure 4).

(1) Construct the graph structure of the street network by modeling the street segments and the street intersections as edges and nodes, respectively. Based on this graph, each point of a fire station is projected to its nearest location on the street network according to the Euclidean distance.
(2) Densify the street network by adding equidistant points on the street segments, and calculate the network kernel density values of the fire incident points on these sampled locations according to Equations (1) and (2). Then, the geometric length and the fire risk of a street segment are integrated into the cost measure of an edge according to Equation (3).
(3) Expand the network from the locations of fire stations. This step generates a network-expanding operator for each fire station, and all the operators would expand along the network simultaneously; the nodes that are traversed by an operator would be assigned as the part of the service area of the corresponding fire station (Figure 4a).
(4) Perform Step (3) repeatedly until one of the operators reaches the boundaries of Golden 5 min coverage.
(5) Stop the network-expanding operators that reach the Golden 5 min boundaries of fire stations, and continue to perform the other operators (Figure 4b).
(6) Perform all of the operators synchronously when they reach the Golden 5 min boundaries (Figure 4c).
(7) Generate the service area of fire station which consists of the nodes and segments traversed by the corresponding expansion operator (Figure 4d).

Compared to the traditional algorithm, our algorithm obtains the service areas in the form of segments and nodes. The proposed algorithm not only can introduce the constraints of the street network and the fire risk into the Voronoi cost measure but also can take into account the specific application of fire rescuing that aims at increasing the coverage area of 5 min in the constructed Voronoi cells.

## 3. Data and Study Area

With the rapid development of the economy in the last 30 years, a large volume of rural land space of China has moved to the urban areas, thus bringing massive population in the major cities in China such as Nanjing. The urbanization process also brings about the increase of fire risk in high-density areas. In the built environment of China, for the effective use of urban space, buildings tend to be closely connected, which usually occupy fire exits [35]. In recent years, urban fire accidents have become more and more costly and this situation has attracted great attention from urban managers. The Chinese government attaches great importance to the impact of fire accidents and has further revised the national fire control law in 2019 [36].

Our study area is located in Nanjing, capital of the Jiangsu province. As a central city, Nanjing is one of the most important scientific research and education bases and comprehensive transportation hubs in the east of China. It has jurisdiction over 11 districts with a total area of 6587 square kilometers. Nanjing is the only megalopolis in the Yangtze river delta of east China. According to the National Bureau of Statistics of China, the average population density of Nanjing is about 1034 persons/km^2^, which is 7.2 times the national average in 2017. Such a high density of population brings enormous pressure on fire risk prevention in Nanjing, which is also faced by other cities in the Jiangsu province. Hence, in recent years, Jiangsu province has promulgated the Fire Safety Index System of Jiangsu Province [37], which is the first national fire work evaluation system in the name of a provincial government. Such policy specifies that the fire accident rate of 10,000 people should be lower than 2 and that the fire death rate should be lower than 0.019. Therefore, although Nanjing faces an increased risk of fire accident in recent years, it acts as a pioneer in the field of fire control in China.

**Figure 5:**
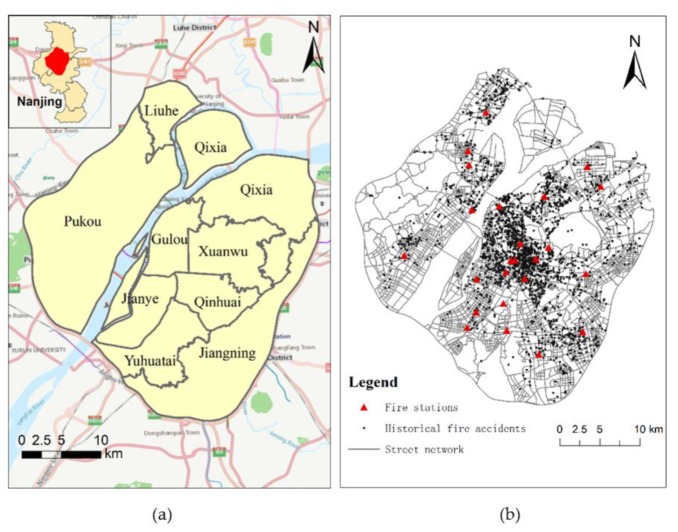
The study area (**a**) and data (**b**): the road network, fire stations, and historical fire incident data.

This research selects the nine most densely populated urban districts of Nanjing as the study area, including Liuhe, Qixia, Pukou, Gulou, Xuanwu, Jianye, Qinhuai, Yuhuatai, and Jiangning (Figure 5a). Our data is obtained from the Fire Department of the Public Security of Nanjing, which contains street network data, fire station data, and fire incident data. More specifically, there are 8636 edges/links and 6248 street intersections/nodes in our street network data. We obtain 24 fire station points and 2503 historical fire accident points from March to December 2015 (Figure 5b).

## 4. Results and Analysis

### 4.1. Impact of Parameters

**Figure 6:**
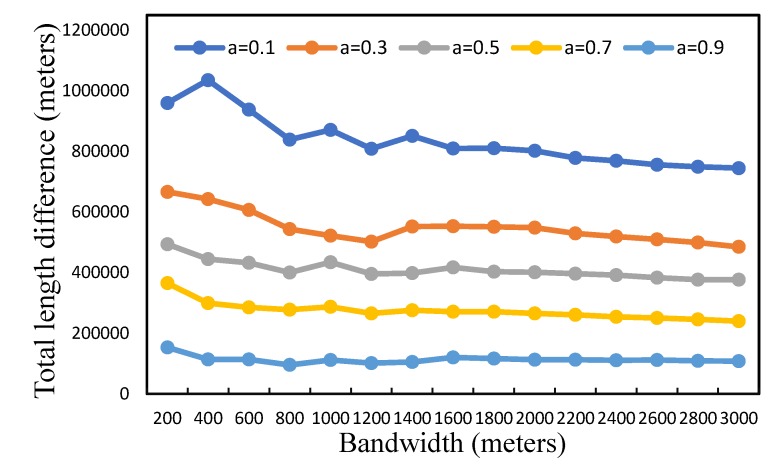
The difference between the service areas delimited by our method and the general Voronoi method with different bandwidths: *a* is the parameter for comparing geometric distance to fire risk in the cost measure of street segment.

**Figure 7:**
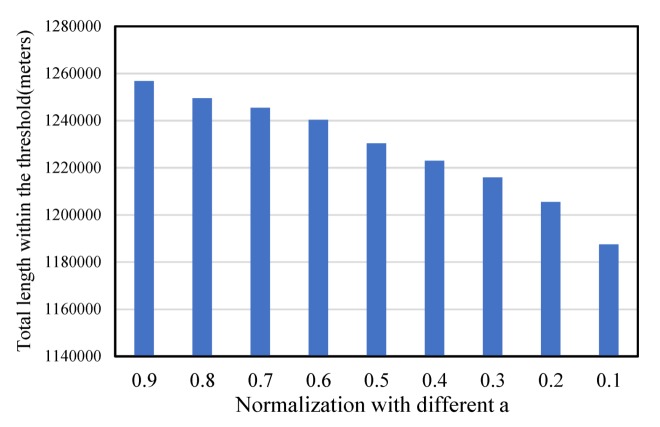
The 5-min coverage length of service areas of fire stations in our result by using different weights (a) for geometric distance and fire risk.

This section discusses the important parameters mentioned above and tries to analyze their impacts on our results of service area delimitation. Since our method uses the network kernel density method to quantify the fire risk across space, it is important to evaluate the impact of the bandwidth on the service area delimitation. In our experiments, we term the network Voronoi diagram without fire risk constraint as N-VD, and then, we compared the N-VD result with our C-VD result, as presented in Figure 6. It can be observed that the sum of the length of different service areas generated by our C-VD and the general N-VD shows a decreasing trend for an increasing bandwidth. With a smaller bandwidth, the difference of service areas decreases more rapidly, and when the bandwidth is larger than 1500 m, the difference between our results and the N-VD result does not change much. The reason may be that, the smaller the bandwidth is, the more details the kernel density distribution has. A large bandwidth emphasizes the global distribution of point density and smooths the local differences of fire risk. Hence, in order to produce stable results, we chose a bandwidth of 1500 m for the following experiments. In addition, for integrating the two factors geometric distance and fire risk (Equation (3)), the weight of a determines the degree to which the accessibility or workload affects the service area division of fire stations. With a larger value of a, the service area division depends more on the accessibility, while with a smaller value of a, our result depends more on the variation of workload across space. In order to evaluate our results with different weights, we calculated the total length of the network segments that could be covered by the fire service areas within 5 minutes. As presented in Figure 7, the length of the 5-min coverage area decreases with decreasing weight a. The reason may be that a small value of a increases the weight of fire risk in the cost measure of street segments, and thus, the fire stations that have high fire risk in their neighborhoods are hard to expand outwards to obtain a large service area in the Voronoi structure. In our experiments, to balance the contributions of geometric distance and fire risk, we chose the weight a = 0.5.

### 4.2. Results and Analysis

**Figure 8:**
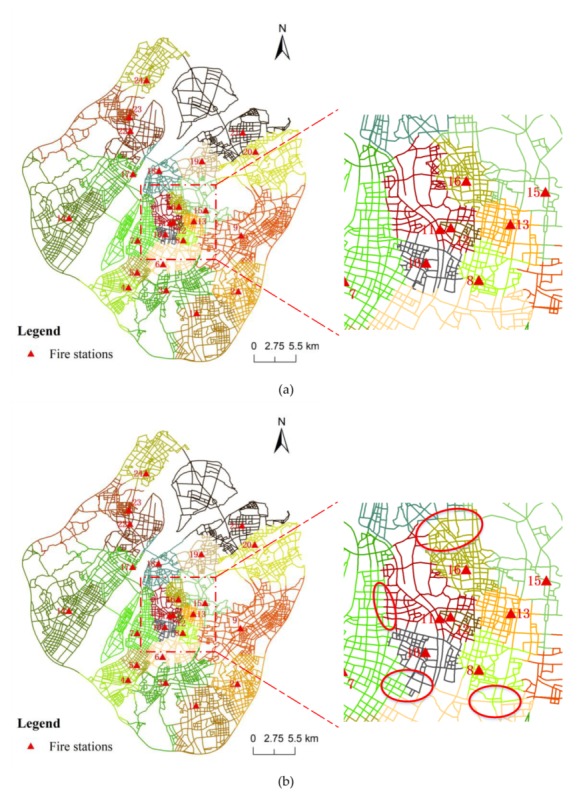
The results of service area delimitation of fire stations using the constrained Voronoi diagram: (**a**) the service area division without Golden 5 min constraint and (**b**) the service area division with Golden 5 min constraint.

Based on the fire records from March to December 2015, our next experiment compared the result of C-VD without the Golden 5 min constraint to that with the Golden 5 min coverage constraint. First, as presented in Figure 6a, even without the Golden 5 min constraint, our C-VD method can still generate reasonable service areas for fire stations, which take into account the accessibility constraint and the workload constraint. The emergency facilities located in the central areas have larger workloads in their neighborhoods, and thus, their service areas become more compact in the tessellation space. In addition, with the introduction of the Golden 5 min constraint, the service areas become more balanced in terms of geometric length. In Figure 8a, some locations are assigned to a facility with travel cost larger than 5 min, while in Figure 8b, they are assigned to a facility with travel cost less than 5 min (please see the locations marked by red ellipses). Our algorithm based on the 5-min threshold of fire-fighting time would help increase the proportion of locations in C-VD that can be reached by fire stations within effective time, and in this way, it can improve the efficiency of fire rescuing.

**Figure 9:**
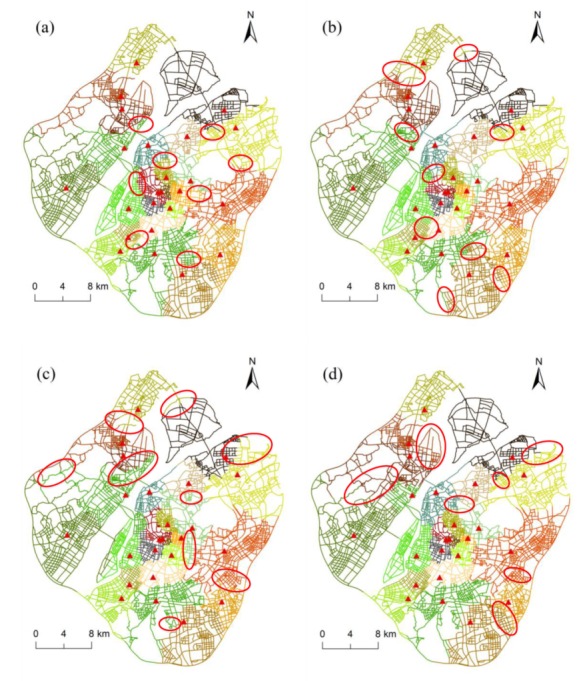

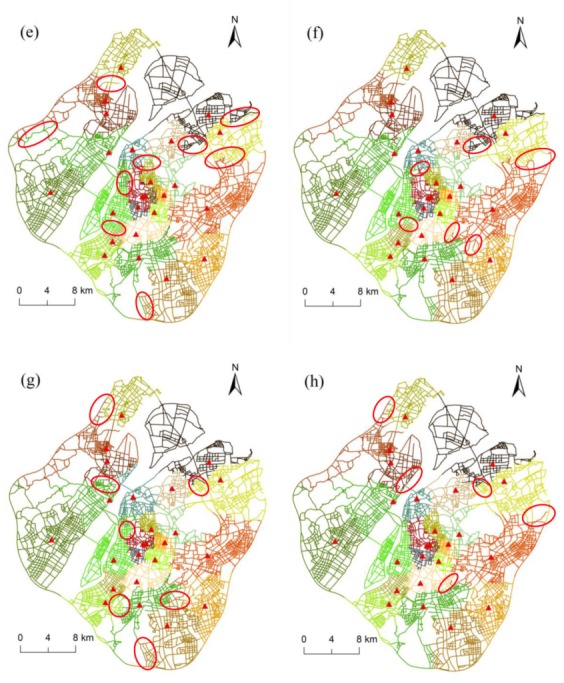

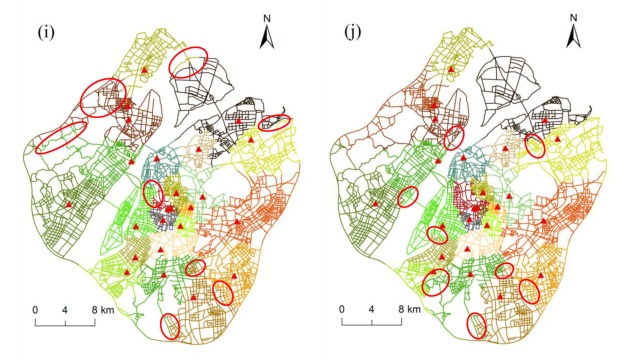
Dynamic service areas of fire stations by using monthly fire incident data from March to December 2015: (**a**) March, (**b**) April, (**c**) May, (**d**) June, (**e**) July, (**f**) August, (**g**) September, (**h**) October, (**i**) November, and (**j**) December. In each subgraph, the red ellipses highlight the difference between the results of this month and the results of the whole period (Figure 8b).

Since fire risk may vary across time, the service area division with addition of fire incident data can be implemented for different time periods. Compared to the static result for a fixed time range, the dynamic results are more suitable for the environments that have significant temporal features. In addition, many applications need to allocate resources according to the change of fire risk across time, and thus, the dynamic service area division may be very valuable for these contexts. Specifically, in the study region, both the frequency and spatial distribution of fire incidents have time dependence. In this respect, we applied our method to generate dynamic service areas of fire stations by using monthly fire incident data. As presented in Figure 9, compared to the result in the whole time period, some significant changes of service area across the time slices are marked using the red ellipses. It can be observed that the maximum change of service area across the time slices is in the central areas of the city. These areas have a relatively high density of fire incidents, and the associated fire risk changes more frequently with time.

**Figure 10:**
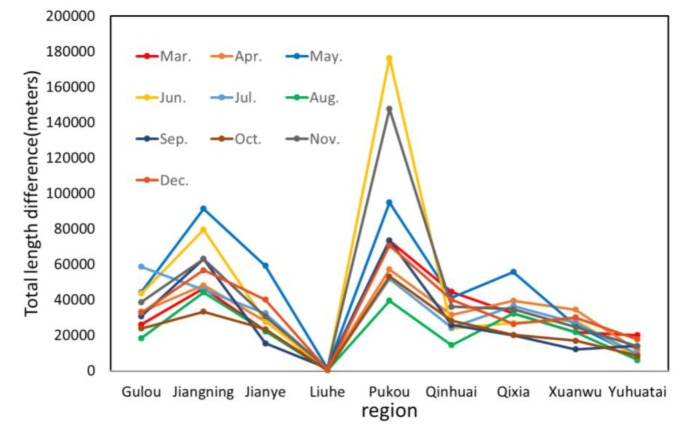
The total length of street segments which are assigned to different fire stations in administrative regions across the time.

In addition, based on the results generated from March to December 2015, we obtained the statistics of change of service area division for different administrative regions, as presented in Figure 10. It can be observed that, in all the months, the trend of service area change is similar for different regions. The service area division in Pukou and Jiangning experienced great changes during the whole time period, while those in Liuhe and Yuhuatai were relatively stable. It indicates that the workloads in Pukou and Jiangning change rapidly across most of the time and that managers should adjust their fire services according to the changing environment.

Next, we obtained the statistics of the service path length for each fire station (Table 1). It can be observed that the two fire stations of No.9 and No.14 have the longest service areas in most of the time. It indicates that the challenge for the two fire stations is to drive a longer distance to cover all the demands. As a comparison, the fire station of No.12 has the shortest length of fire service area. Hence, there may be high fire risk in the neighborhood of this fire station, and the main challenge is to handle the frequent fire accidents in the narrow region. In addition, as presented in Figure 8, the fire station of No.12 is surrounded by many fire stations and the geometric restriction can also contribute to the small service area.

### 4.3. Evaluation and Discussion

**Figure 11:**
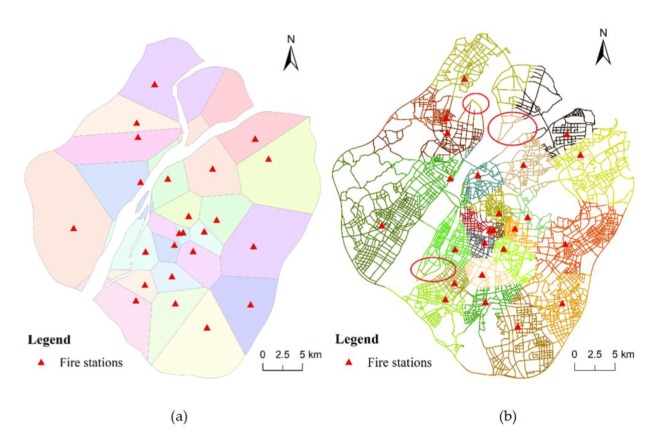
The service area division generated by the traditional planar Voronoi diagram: (**a**) the two-dimensional Voronoi structure and (**b**) the street segments that fall into each two-dimensional service area.

First, we compared the result of C-VD to that of the traditional planar Voronoi diagram, as presented in Figure 11. Both the planar method and C-VD can generate a complete and nonoverlapping division of the entire study region. However, the service area generated by C-VD is constrained to the street network, while that by the planar Voronoi method is an equidistant division in the two-dimensional plane. In addition, we partitioned the street network according to the service area division generated by the planar Voronoi diagram (Figure 11b). It can be observed that the street segments in a single service area are not always contiguous, and there exist enclaves in the service area division (please see the segments marked by red circles). This means that this fire station may need to pass through the service area of other fire stations to reach the destination. In this respect, the noncontiguous segments do not meet the continuity of service area and may hinder fire rescuing efficiency. As a comparison, the result generated by our C-VD is based on the actual route directly, and thus, it can maintain the continuity of service areas of fire stations. Another advantage of preserving the continuity of service areas is that the route from any location in a service area to the corresponding fire station can be clearly presented.

**Figure 12:**
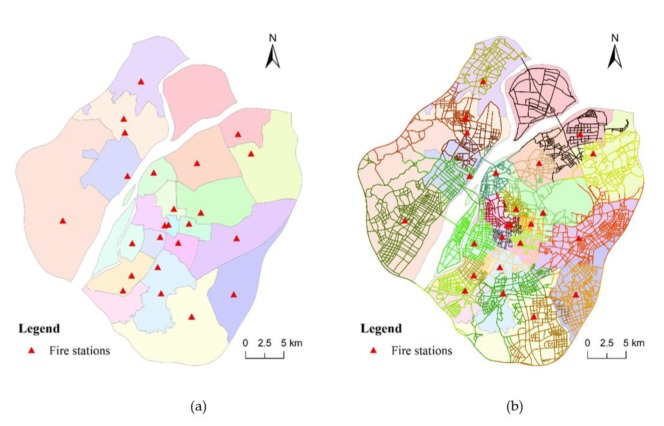
The official division of service area of fire stations (**a**) and the overlap of the result generated by our method and the official result (**b**).

Then, we compared our result to the official division of service area of fire stations (Figure 12a), which are based mainly on the administrative division. In order to present the difference between the official division and our division clearly, we overlap the two divisions as presented in Figure 12b. We can find that, although the official plan is also based on the two-dimensional plane like the traditional planar Voronoi diagram, it can preserve the continuity of service area. In addition, the official division is closer to the division generated by our C-VD than the planar Voronoi division. However, there are two issues that should be paid attention to: (1) as presented in Figure 12a, there are some service areas, each of which contain two or more fire stations; (2) some service areas do not have fire stations. These issues could result in difficulty of task allocation in practical fire rescuing.

**Figure 13:**
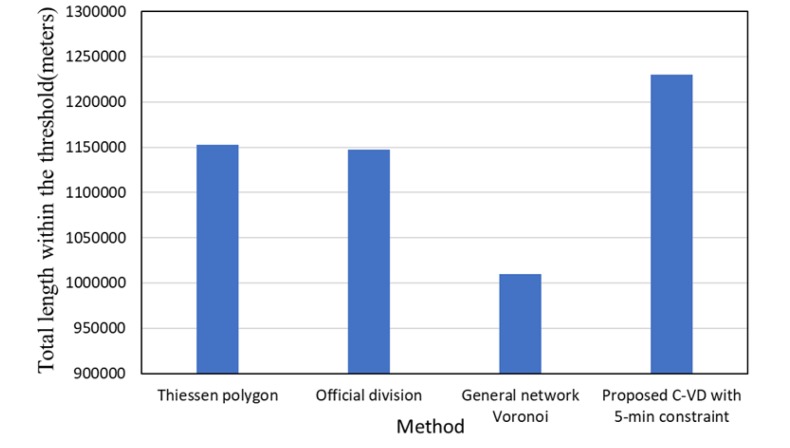
The street segment length of service areas within the Golden 5 min threshold obtained by different methods.

**Figure 14:**
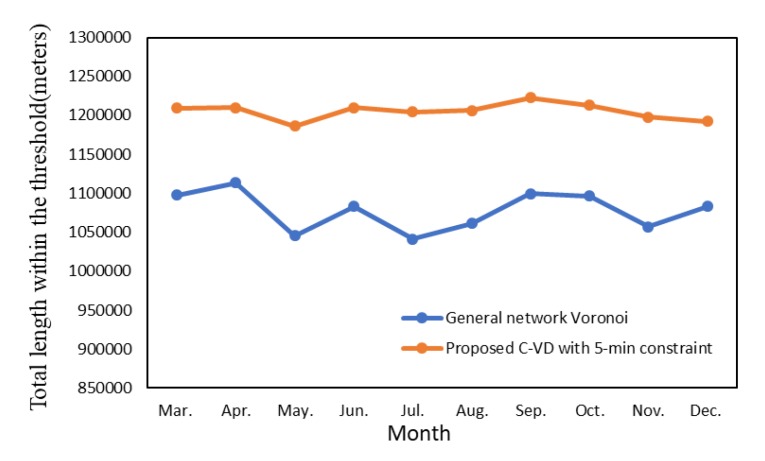
The street segment length of service areas within the Golden 5 min constraint in different time slices.

Since fire rescuing aims at reaching the destination within 5 min, we then compared the 5-min coverage length in the service areas generated by the traditional Thiessen polygon method (i.e., planar Voronoi method), the official division method, the general network Voronoi method without the Golden 5 min constraint, and the proposed C-VD with the Golden 5 min constraint. The Thiessen polygon method and the official division method are based only on the geometric distance in 2D plane, and thus, they obtain a larger 5-min coverage length than the general network Voronoi method, which takes into account both the network distance and the spatial variation of fire risk (Figure 13). With addition of the Golden 5 min constraint, our proposed C-VD can significantly increase the effective coverage area in the constrained Voronoi structure. It generates the largest 5-min coverage area among all the results. Furthermore, in order to examine the effectiveness of our method in different time slices, we also obtained the statistics of monthly C-VD. As presented in Figure 14, the 5-min coverage length of C-VD is always larger than that of the general network Voronoi diagram during the whole time period.

Through the above comparative analysis, we can draw a conclusion that the proposed C-VD provides a reasonable service area partitioning scheme for emergency facilities by adding street network data and fire incident data. The traditional planar method is hard to incorporate both of the constraints accessibility and workload, and that limits its application in practical emergency events. However, although the network Voronoi diagram inherits the merit of the general Voronoi diagram that partitions the study region equally, it takes little account of the Golden 5 min constraint of fire rescuing. Thus, we further improve the network Voronoi diagram by introducing a balance strategy into the network expansion operation. More specifically, during the process of constructing the network Voronoi diagram, the expansion operators of the generator that reach the distance equal to 5 min would be stopped until the other operators reach the 5-min boundaries. In this way, besides the addition of workload, the proposed method can also ensure the efficiency of emergency rescuing in fire events.

All of the above results are based on two assumptions. One is that the distance from the fire station to the street is ignorable with regard to the whole street network, and the other is that the service area of fire stations is constrained by a threshold of 5 minute. We have conducted two additional experiments to test the validity of the hypothesis.

### 4.4. The Effect of Projection Distance

In the previous experiment, we directly projected the fire station onto the street network and used its projection point as the starting generator but neglected the distance from the original location to the projection point. There are two main reasons for ignoring such distance. First, in the data we obtained, the fire station locations are recorded as points. Although all fire stations have a certain distance from the road, this distance effect would be roughly counteracted since the Voronoi diagram is constructed as a competitive relationship between generators. Second, the locations of the fire stations are quite close to the road network. Compared with the network distance between two fire stations, the distance from the fire station to its projection point is so small that it can be ignored in C-VD.

**Figure 15:**
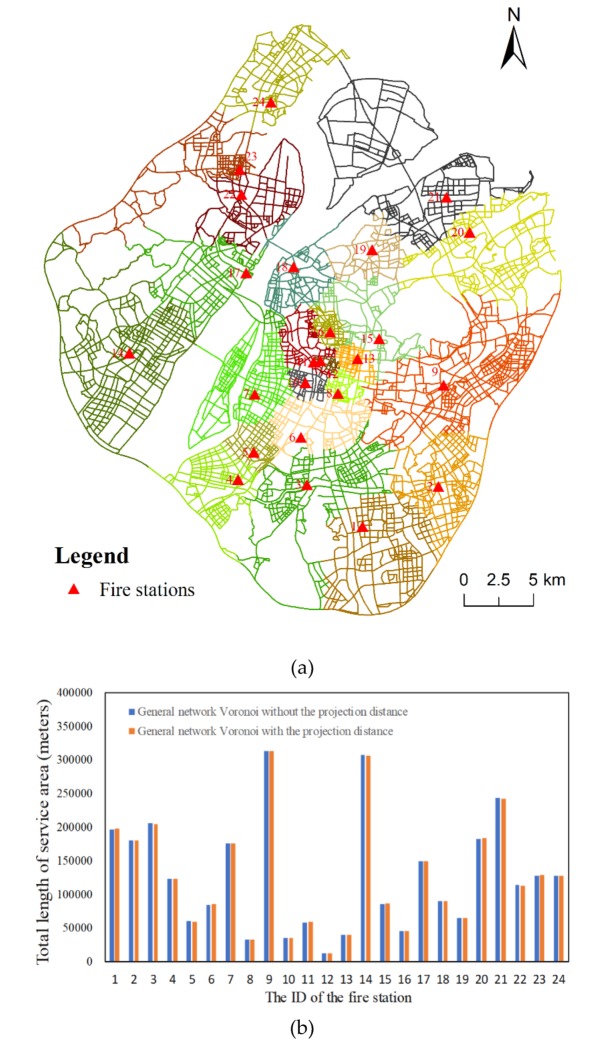
The result of service area delimitation of fire stations using the general network Voronoi diagram with projection distance (**a**) and the difference between the results constructed without projection distance and with projection distance (**b**).

**Figure 16:**
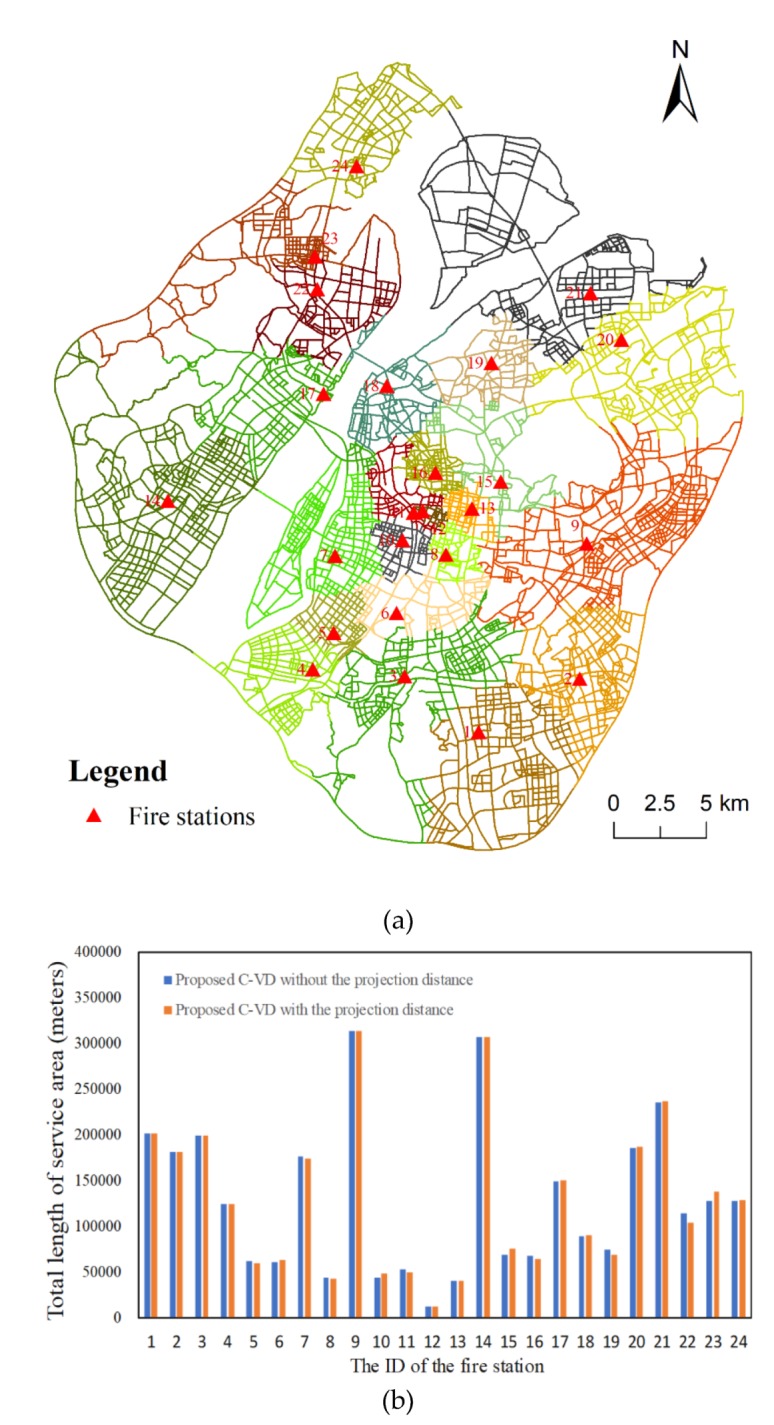
The result of service area delimitation of fire stations using the C-VD with projection distance (**a**) and the difference between the results constructed without projection distance and with projection distance (**b**).

Nevertheless, we also verified the impact of this projection distance on the C-VD. Specifically, when constructing the general network Voronoi diagram and the proposed C-VD with a 5-min constraint, we firstly measured the projection distance as the distance from each projection point to the network and then expanded these generators to obtain a Voronoi diagram as described in Section 2.3. For the general network Voronoi diagram, we can barely see the difference between the original result without projection distance (Figure 8a) and the result with projection distance (Figure 15a). Then, we calculated the length of the service area of each fire station under the two conditions (Figure 15b). The result shows that there are some very tiny changes to the service areas of the fire stations, e.g., fire station No. 5, No. 6, No. 21, No. 22, and No. 23. Furthermore, compared with the C-VD without projection distance (Figure 8b), the C-VD with projection distance also shows small changes (Figure 16a). We calculated the statistics of these projection distances of fire stations and found that most of these projection distances approximately equal to 30 m. Since the Voronoi diagram is a reflection of the competitive relationship among fire stations, such small differences would not have a large impact on our result (Figure 16b).

### 4.5. The Effect of Different Thresholds

**Figure 17:**
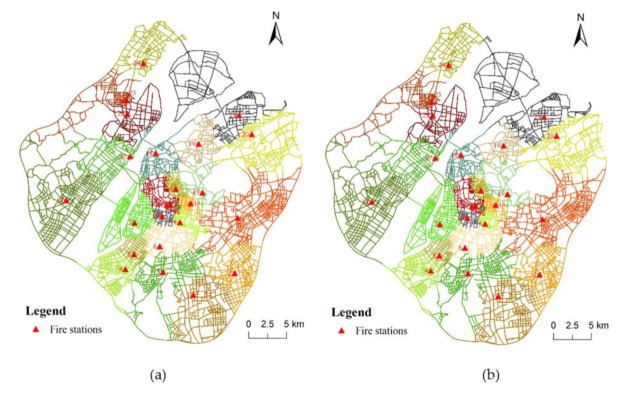
The result of service area delimitation of fire stations using the proposed C-VD with 10-min constraint (**a**) and 15-min constraint (**b**).

In order to evaluate the behavior of our method with different travel cost thresholds, we extended the time requirement using the thresholds of 10 min (Figure 17a) and 15 min (Figure 17b). Comparing with Figure 8b, we find that the service area of some fire stations has changed significantly. Specifically, such change increases with the increase of the threshold. For example, in Figure 17b, the service area of fire station No. 19 is significantly smaller than that in Figure 17a. The reason is that, with a relatively small threshold, the C-VD would be largely constrained by the time threshold but that, with a relatively large threshold, the C-VD would depend mainly on the original neighboring relationships of fire stations. Some of the travel costs between fire stations are larger than 5 min but most of them are less than 15 min, and that means that different travel time thresholds have different impacts on the C-VD.

**Figure 18:**
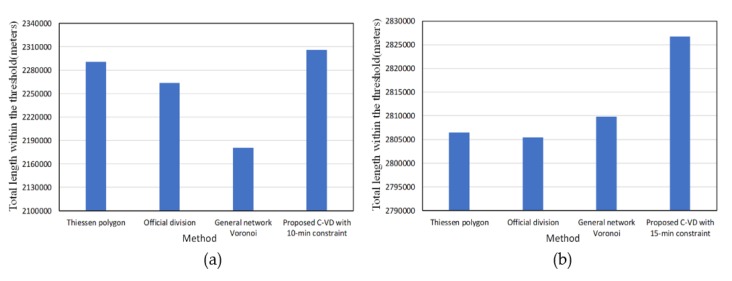
The street segment length of service areas within the 10-min threshold (**a**) and the 15-min threshold (**b**) obtained by different methods.

In addition, as presented in Figure 18, we compared the results of the traditional Thiessen polygon method, the official division method, the general network Voronoi method, and the proposed C-VD based on different time constraints. When the threshold value is 10 min, the statistics (Figure 18a) do not change much with respect to the result based on the threshold value of 5 min (Figure 13). When the threshold is 15 min, the difference generated by the four methods becomes smaller (Figure 18b). Nevertheless, no matter which time threshold we take, the C-VD can serve the largest area within the time threshold.

## 5. Conclusions and Future Work

Emergency services are closely related to people’s lives and property in built environments. How to delimit the service areas of emergency facilities effectively is a critical component in this process. However, due to the lack of relevant event data, research of emergency facilities mainly focuses on the general computing method, and there is little research on the investigation of service area delimitation of emergency facilities by combining the actual emergency rescuing constraints. In our work, we obtained the data sets of fire stations, street network, and historical fire events in Nanjing, and with these data, a new method, the constrained Voronoi diagram (C-VD), is proposed to delimit the service areas of fire stations by considering the constraints of accessibility, fire risk, and coverage area.

The proposed C-VD provides an effective service area division of fire stations through adding three constraints to the traditional planar Voronoi diagram. First, since the movement of vehicles in urban areas depends on the alignment of street network, we propose to use the network shortest-path distance rather than Euclidean distance to analyze the neighboring relationships between locations. This is particular useful for managing emergency facilities (e.g., fire stations) because the network route is the main transport for fire engines. In this respect, we obtain the service area as a set of street segments and intersections, which can facilitate the path planning of fire rescuing on the basis of our result. Second, besides the geometric distance, the variation of fire risk across the space can also impact the service area partitioning of fire stations. In general, the higher fire risk a place has, the more fire protection resources should be allocated to this place. Under our context, this constraint would result in a fire station near high-risk locations having a relatively small service area for the effective management of fire events. Considering the distance decay effect of fire risk, our proposed method calculates the network kernel density values of historical fire event points to quantify the fire risk along street network. Hence, the service areas delimited by our C-VD can take into account the spatial variation of demand in fire rescuing. Finally, in order to introduce the constraint of fire rescuing that fire engines should arrive at the location of fire event within 5 min, the proposed C-VD extends the traditional network expansion operation by increasing the 5-min coverage areas in a Voronoi tessellation. Since fire risk changes across time and space, our method can generate dynamic service area division with the addition of fire events data in different time periods. Such dynamic result may be very valuable for the applications that need to allocate rescuing resources according to time. Actually, the occurrence of fire events in urban areas has a significant characteristic of “time dependence” (please see Figure 9).

In addition to the abovementioned issues, there are more constraints to consider in the service area division of fire services. For example, although we have considered the network distance in the spatial analysis, the constraints of traffic congestion, multiple lanes, and road direction are not added [38,39]. In the future, we plan to obtain the actual trajectory data of fire engines to estimate the travel time on different routes. Besides, with more semantic properties of fire stations (e.g., number of firefighters and number of fire engines), we can improve the effectiveness of our result further. In this way, the relationship between supply and demand can be better modeled in our proposed C-VD with the Golden 5 min constraint.

## Figures and Tables

**Table 1:** The fire stations that have the longest and shortest service lengths in different time slices.

	The Longest Services Areas		The Shortest Services Areas	
Month	Fire Station ID	Length (m)	Fire Station ID	Length (m)
Mar.	14	327,036.66	12	22,247.96
Apr.	14	324,476.68	12	5614.32
May.	9	329,084.45	12	13,652.2
Jun.	9	349,962.72	12	20,544.32
Jul.	9	345,481.59	12	31,356.93
Aug.	9	351,839.45	12	11,471.45
Sep.	14	324,335.48	12	11,364.70
Oct.	9	318,165.59	12	12,147.06
Nov.	9	311,006.02	12	9315.43
Dec.	9	300,725.39	12	16,042.00
